# Phenolic Compositions and Antioxidant Activities Differ Significantly among Sorghum Grains with Different Applications

**DOI:** 10.3390/molecules23051203

**Published:** 2018-05-17

**Authors:** Shuyu Shen, Rui Huang, Charlie Li, Wenyan Wu, Honglin Chen, John Shi, Shiguo Chen, Xingqian Ye

**Affiliations:** 1 Zhejiang Key Laboratory for Agro-Food Processing, Fuli Institute of Food Science, College of Biosystem Engineering and Food Science, Zhejiang University, Hangzhou 310058, China; vera_shensy@163.com (S.S.); ruihuang1995@163.com (R.H.); 21713081@zju.edu.cn (W.W.); chenhlys@sina.com (H.C.); 2 Department of Environmental Toxicology, University of California-Davis, Davis, CA 94564, USA; csjli@ucdavis.edu; 3 Guelph Research and Development Center, Agriculture and Agri-Food Canada, Guelph, ON N1G 5C9, Canada

**Keywords:** sorghum grain, phenolic composition, antioxidant capacity, brewing sorghum, taxifolin

## Abstract

Sorghum grains with different applications had different phenolic profiles, which were corresponded to various antioxidant capacities. In this study, total phenolic, proanthocyanidins and flavonoids contents, as well as contents of individual phenolic compounds from sorghum grains with various applications were determined, and their antioxidant capacities were evaluated. Total phenolic contents (TPC) and total proanthocyanidins contents (TPAC) showed strong correlation with antioxidant activities (*r* > 0.95, *p* < 0.01). Hongyingzi (S-1), one of the brewing sorghums, showed the highest level of TPC and TPAC, while white grain sorghum (S-8) had the lowest. Except for black grain sorghum (S-7), that contained the highest contents of ferulic acid, brewing sorghum grains contained the higher contents of the most individual phenolic compounds, especially the variety S-1. The correlation among individual phenolic compounds and antioxidant activities indicated that the free forms of protocatechuic acid (*r* = 0.982 of FRAPassay, *p* < 0.01) and taxifolin (*r* = 0.826 of FRAP assay, *p* < 0.01) may be the main functional compounds. These results indicate that brewing sorghum grains can also be utilized as effective materials for functional foods.

## 1. Introduction

Sorghum (*Sorghum bicolor* (L.) Moench) is the fifth most important crop after wheat, rice, maize and barley in the world [1] and plays a vital role in Asia, Africa and other semi-arid regions [2].

Sorghum can be processed and applied to various fields. In general, whole grain sorghum is boiled like rice, roasted or popped like corn and its flour is used to make porridge, pancake, bread, dumplings, beers or fermented beverages. For example, in Africa, sorghum grain is grounded and fermented for beer production. In India, sorghum flour is used to make bread. In Korea, sorghum is cooked with rice and its flour is used to make cake. In China, sorghum is fermented and distilled to produce spirit, maotai, which is regarded as one of the country’s most famous liquors [3].

Sorghums for different usages come from different varieties. White grain sorghum is often used in cooking, while red and brown grain sorghums with more bioactive compounds, such as tannins, are normally used for beer making [3]. These compounds are considered to be beneficial to human health and are widely applied in many processed foods as functional ingredients [4]. In vitro and in vivo studies have demonstrated that active compounds isolated from sorghum, mainly phenolics, promote beneficial changes in parameters related to noncommunicable diseases such as obesity, diabetes, dyslipidemia, cardiovascular disease, cancer, and hypertension [2]. Therefore, the development of sorghums should be reconsidered for their bioactive compounds.

Phenolic compounds in sorghum are mainly classified into phenolic acids, proanthocyanidins and flavonoids. It was reported that the genotype of the sorghum and the environment where they were grown could affect their color, appearance and nutritional quality, which contributed to the differences of their phenolic compounds [5]. Studies showed that varieties with pericarp and black testa had three to four times more total 3-deoxyanthocyanidins than red and brown varieties [6]. The main flavanones in sorghum were found to be the lowest in white varieties and those with lemon-yellow pericarp had the highest contents [7].

The objective of the present study is to identify and quantify phenolic compositions in sorghum grains with different applications and evaluate their antioxidant capacities. The study is to reveal the correlation between phenolics and sorghum varieties and to provide useful information for exploring the nutritional value of sorghums.

## 2. Materials and Methods

### 2.1. Samples

**Figure 1:**
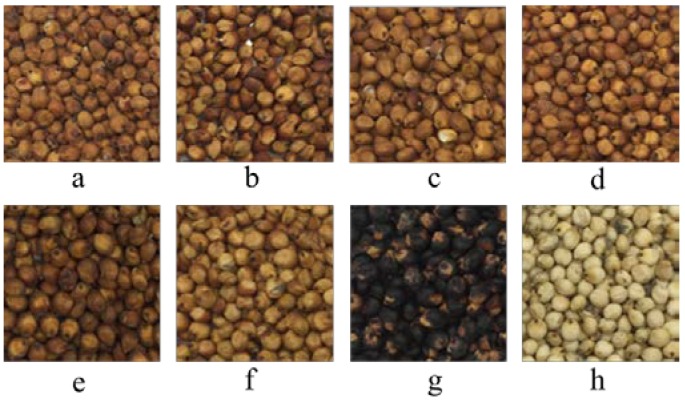
Pictures of the investigated sorghum grains. Letters (**a**–**h**) represent grains of S-1, S-2, S-3, S-4, S-5, S-6, S-7 and S-8, respectively.

Eight varieties of sorghum grains (Figure 1) for different applications were selected for analysis, including four brewing sorghum varieties, Hongyingzi (S-1), Hongzhenzhu (S-2), Dongbei sorghum (S-3) and Jiangsu sorghum (S-4), two sorghums for seed reserving, Jiliang 2 (S-5) and Longza 11 (S-6), one black grain sorghum (S-7) and one white grain sorghum, Longmi sorghum (S-8), as a staple food and meanwhile as a comparison, were all provided by Shandong Academy of Agricultural Science (Jinan, China). The detailed geographic information of the analyzed sorghum grains was showed in Table 1. All samples were ground to achieve a particle size that passed through a 500-μm sieve and the moisture of all samples was determined and was around 13%.

### 2.2. Chemicals and Reagents

High-performance liquid chromatography (HPLC) grade, methanol, 2,2-diphenyl-1-picrylhydrazyl radicals (DPPH), 2,4,6-tris(2-pyridyl)-*S*-triazine (TPTZ), naringenin and apigenin were purchased from sigma-Aldrich (St. Louis, MO, USA). Apigeninidin, luteolinidin were purchased from J&K (Beijing, China). Taxifolin, luteolin, rutin, protocatechuic acid, caffeic acid, *p*-coumaric acid, ferulic acid and ascorbic acid was obtained from Aladdin (Shanghai, China). HPLC-grade acetonitrile was purchased from Merck KgaA (Darmstadt, Germany). All the other reagents of analytical grade were bought from Sinopharm Chemical Reagent Co., Ltd. (Shanghai, China).

### 2.3. Sample Preparation

Free phenolics were extracted, by the modification of the method as explained by Adom and Liu [8] with small modifications. Briefly, 2.0 g of each sample was extracted with 30 mL chilled ethanol (80%) for 15 min followed by centrifugation at 2500× *g* for 10 min. The supernatant was removed, and residue was re-extracted twice. Supernatants were pooled, evaporated at 45 °C to dryness and reconstituted to 10 mL with methanol. Each sample was extracted in triplicate and extracts were stored at −40 °C until analysis. 

Bound phenolics were extracted, following the method as explained by Adom and Liu [8] with small modifications. In short, the residues in triplicate, obtained after the extraction of free phenolics, were digested using 30 mL of 2 M sodium hydroxide at room temperature for 1 h with shaking. Then, the mixture was neutralized with concentrated hydrochloric acid and extracted with hexane to remove lipids. The remaining mixture was extracted five times with ethyl acetate. The ethyl acetate fraction was evaporated at 45 °C to dryness and reconstituted to 10 mL with methanol. The extracts were stored at −40 °C until analysis.

### 2.4. Determination of Total Phenolic Contents

Total phenolic contents (TPC) was determined using the Folin-Ciocalteu assay [9,10] adapted to 96-well microplates. Firstly, samples were diluted appropriately with ultrapure water. A 450 μL ultrapure water was transferred to 2 mL centrifuge tube, followed by 100 μL diluted samples or standards, and mixed with 100 μL of 2-fold-diluted Folin-Ciocalteu reagent. After 6 min, an aliquot of 600 μL of a saturated sodium carbonate solution (75 μg/L) was added to the mixture and react at room temperature. After 120 min, 250 μL aliquots were transferred to microtiter plates and the absorbance was measured at λ = 760nm using a microplate reader (Thermo Fisher Scientific Oy Ratastie 2, Vantaa, Finland). Samples without reaction reagent which was substituted by the same volumn of ultrapure water were used as blank. TPC were calculated on the basis of standard curves and expressed as mg gallic acid equivalents (GAE)/100 g grain (dry weight, DW).

### 2.5. Determination of Total Flavonoid Contents

Total flavonoid contents (TFC) was determined by aluminum chloride method [11,12] adapted to 96-well microplates. A 150 μL solution of appropriately diluted sample solutions was separately mixed with 0.45 mL methanol, 30 μL of 10% aluminum chloride, 30 μL of 1 M potassium acetate and 2.8 mL of deionized water and left at room temperature for 30 min. The absorbance of the reaction mixture was measured at 415 nm. TFC were calculated based on the standard curves, and the final values were expressed as mg rutin equivalents (RE)/100 g grain (dry weight (DW)).

### 2.6. Determination of Total Proanthocyanidins Contents

Total proanthocyanidins contents (TPAC) was determined by acid-butanol assay [13,14]. Briefly, 6 mL of n-butanol + conc. HCl (95:5 *v*/*v*) and 1 mL sample extract were added to a glass tube. After mixing with 0.2 mL of 2% (*w*/*v*) NH^4^Fe^III^(SO_4_)^2^ (in 2 mol/L^−1^ HCl), the tube was then placed in boiling water bath for 45 min. The absorbance was recorded at 550 nm after the tube cooling down to room temperature. Blank spectra were obtained for each extract before boiling. TPAC were calculated based on the standard curves, and the final values were expressed as mg cyanidin chloride equivalents (CCE)/100 g grain (DW).

### 2.7. Individual Phenolics Separated by High-Performance Liquid Chromatography

Phenolic compounds contents, including both extractable and bound phenolic compounds, were analyzed on a Waters system with UV-Vis detector. A reverse phase column (ZORBAX SB-C18, 250 mm × 4.6 mm i.d., 5 μm) was used to perform chromatographic separation. The detection wavelengths were set at 288 nm and 360 nm. Column temperature was maintained at 25 °C. Injection volumn was 10 μL. The mobile phase consisted of 0.1% formic acid in LC-MS grade water (*v*/*v*) (solvent A) and LC-MS grade acetonitrile (solvent B). The solvent flow rate was 0.5 mL/min with the flowing linear gradient elution: 5~15% B (5 min), 15~50% B (40 min), 50~70% B (2 min), 70~100% B (1 min), 100% B (7 min), 100~5% B (1 min), 5% B (9 min) [15]. Individual phenolic compounds concentration of extracts was calculated from each standard calibration curve and expressed as mg phenolics/100 g grain (DW). 

### 2.8. Individual Phenolics Identified by RP-UHPLC-QTOF/MS

The identification of individual phenolic compounds was analyzed on a Waters UPLC (Waters Corp., Milford, MA, USA) coupled with Mass Spectrometry on a AB Triple TOF 5600 plus System (AB SCIEX, Framingham, MA, USA). The LC separation parameters and column were the same as showed in 3.7. The optimal MS conditions: scan range *m*/*z* 100–2000. Negative ion mode: source voltage was −4.5 kV and the source temperature was 550 °C. The pressure of Gas 1 (Air) and Gas 2 (Air) were set to 50 psi, the pressure of Curtain Gas (N2) was set to 30 psi. The Injection volume was set at 10 μL. The flow rate was 0.2 mL·min^−1^. The Maximum allowed error was set to ±5 ppm. Declustering potential (DP), 100 V; collision energy (CE), 10 V. For MS/MS acquisition mode, the IDA-based auto-MS2 was performed on the 8 most intense metabolite ions, the parameters were almost the same except that the collision energy (CE) was set at −40 ± 20 V, ion release delay (IRD) at 67, ion release width (IRW) at 25. In a cycle of full scan (1 s). The scan range of *m*/*z* of precursor ion and product ion were set as 100–2000 Da and 50–1500 Da, respectively. The exact mass calibration was performed automatically before each analysis employing the Automated Calibration Delivery System.

### 2.9. Determination of Antioxidant Activities

#### 2.9.1. DPPH Free Radical Scavenging Activity

The stable free radical diphenylpicrylhydrazyl (DPPH) assay was performed based on a method by a previous report with slight modification [16]. Briefly, 0.2 mL of diluted samples were added into 2.8 mL of 0.1 mM DPPH in 80% ethanol (water, *v*/*v*). 80% ethanol was used as the reagent blank. The mixture solution was kept at room temperature in the dark for 30 min and the absorbance was measured at 517 nm. Results were expressed as mg ascorbic acid equivalents (VcE)/g grain (DW).

#### 2.9.2. Reducing Power

The ferric reducing ability of plasma (FRAP) assay was determined based on a previous report with slight modification [16]. The FRAP reagent consisted of 10 mM TPTZ (dissolved in 40 mM HCl), 20 mM ferric chloride and 0.1 mol/L acetate buffer (pH 3.6) at a ratio of 1:1:10 (*v*/*v*/*v*). 100 μL of samples reacted with 4.9 mL of prepared FRAP solution for 10 min at 37 °C in the dark, and then the absorbance was measured at 593 nm. The results were expressed as mg VcE/g grain (DW).

### 2.10. Statistical Analysis

All data are expressed as the means ± standard deviation of three replicates. Statistical analysis was performed using SPSS v19.0 software (SPSS for Windows, Release 19.0, SPSS Inc., Chicago, IL, USA). Significant differences among the samples were calculated using one-way ANOVA, followed by Duncan’s multiple-range test at 5% level (*p* ≤ 0.05). Correlation was determined using Pearson’s correlations.

## 3. Results and Discussion

### 3.1. Total Phenolic, Flavonoid and Proanthocyanidins Contents

Free, bound and total phenolic, flavonoid and proanthocyanidin contents, as well as their percentage contribution to the total of eight varieties of sorghum grains (S-1 to S-8, specific information of them were shown in Section 2.1) are summarized in Table 2. On the whole, brewing sorghum grains except S-4 showed higher phenolic compounds than other varieties, while the white grain sorghum, S-8, showed the lowest contents of all.

The TPC showed obvious variations among different varieties ranging from 174.40 ± 4.09 to 1238.83 ± 31.67 mg GAE/100 g grain (Total, DW). Among all the brewing sorghum varieties, S-1 had the highest TPC, followed by S-3 while S-4 had the lowest. The two seed-reserved sorghums contained moderate TPC (559.50 ± 50.39 and 640.67 ± 54.87 mg GAE/100 g grain, DW) compared with other varieties. The grain color is one of the reason that results in the TPC differences among these varieties, however the variation in brewing sorghum grains may come from other reasons, such as the strain and growing environment [5]. S-1 and S-2 were close relatives which were grown at the north of Guizhou Province while S-3 was grown at Dongbei and S-4 was grown at Jiangsu Province. So, S-1 may be a high-quality variety that contains high levels of TPC. In addition, we found that the TPC of free fractions were considerably higher than their corresponding bound fractions. Especially in S-8, free phenolic contents contributed to the total phenolics at 98.07%. However, the results conducted by Wu G and co-investigators [17] showed that there were no significant differences between free and bound total phenolics in three red pericarp sorghum genotypes. It has also been reported that a high proportion of bound phenolics were presented in cereals [8]. These variations might be mostly due to differences in species and varieties, or growth conditions as Wu G and co-investigators [17] discovered that total phenolics changed significantly under two irrigation treatments. Evaluation methods may also be a vital impact factor, especially the various solvents for extraction. We used 80% chilled ethanol as solvent to extract phenolics [8], considering its safety, while methanol was used by others [17,18].

TFC of eight sorghum grains ranged from 11.72 ± 1.69 to 61.10 ± 5.46 mg RE/100 g grain (total, DW). S-1 showed the highest TFC, S-4 and S-5 exhibited relatively high TFC while S-8 showed the lowest TFC, and the other four varieties exhibited not significant differences. The present results were less than the reported levels in sorghums [17] but it was very close to the result conducted by HPLC in this study (Table 3). This might mainly be due to the differences of varieties and analytical methods. Many assays used the NaNO_2_-Al^3+^-NaOH method to determine the TFC [17,19]. However, after adding NaOH, blood red flocculent precipitate was found, which might be due to the formation of complex precipitates by proanthocyanidins with aluminum ions under alkaline environment [20,21]. Thus, the aluminum chloride method was chosen over the NaNO_2_-Al^3+^-NaOH method.

**Figure 2:**
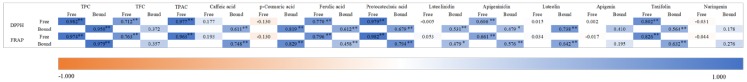
Pearson’s correlation coefficients of total phenolics, total flavonoids, total proanthocyanidins, individual phenolic compounds and antioxidant capacities. Blue color indicates good positive correlation (close to 1), white means low correlation (close to 0) and orange indicates good negative correlation (going to −1). Data with double asterisk (**) and single asterisk (*) are statistically significant at *p* level < 0.01 and *p* level < 0.05, respectively.

In this study, the levels of TPC in free fractions is considerably higher than its corresponding TFC. We determined the total proanthocyanidins contents (TPAC) in free fraction and found extremely high correlation between TPC and TPAC (*r* = 0.991, *p* < 0.01, Figure 2). Therefore, proanthocyanidins may be the big fraction contributed to TPC.

### 3.2. HPLC and UPLC-QTOF/MS Analysis of Phenolic Compounds

**Figure 3:**
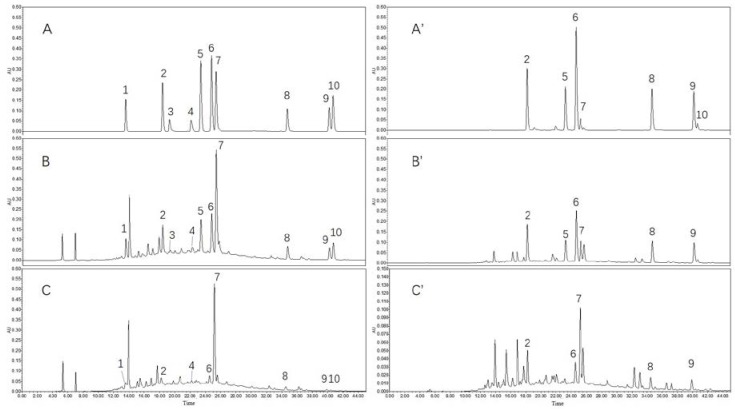
Representative chromatograms of sorghum grain phenolic compounds. (**A**) Standard solution of 10 phenolic compounds standard detected in 288 nm ((**A’**) was detected in 360 nm); (**B**) Free phenolic compounds from variety S-1 with standards detected in 288 nm ((**B’**) was detected in 360 nm); (**C**) Free phenolic compounds from variety S-1 detected in 288 nm ((**C’**) was detected in 360 nm). Peak 1, 2, 3, 4, 5, 6, 7, 8, 9 and 10 are protocatechuic acid; caffeic acid; luteolinidin; apigeninidin; *p*-coumaric acid; ferulic acid; taxifolin; luteolin; apigenin and naringenin, respectively.

Ten phenolic compounds were separated via HPLC separation elution and MS ionization was operated in negative mode. The identification of compounds was based on accurate MS and MS/MS spectra and comparison with literature [15]. The list of tentative identified compounds by RP-UHPLC-QTOF/MS is reported in Table 4 in order of class. The MS/MS information of all the detected phenolic compounds were showed in the Supplementary Materials. The UV absorption chromatograms were shown in Figure 3 and the contents of the evaluated phenolic compounds were listed in Table 3. All the standard phenolic compounds were individually tested through HPLC-DAD system at first by scanning between 200–400 nm. Therefore, protocatechuic acid, *p*-coumaric acid, luteolinidin, apigeninidin, taxifolin and naringenin were detected and quantified in 288 nm; Caffeic acid, ferulic acid, luteolin and apigenin were detected and quantified in 360 nm.

All sorghums contain phenolic acids, which are located in the pericarp, testa, aleurone layer and endosperm [5]. Phenolic acids consist of two classes: hydrobenzoic and hydrocinnamic acids. In the present study, four phenolic acids, namely caffeic acid, *p*-coumaric acid, ferulic acid and protocatechuic acid were quantified in the free and bound extracts of the samples investigated.

Among four brewing sorghum grains and two seed-reserved sorghum grains, the contents of bound form phenolic acids were higher than the contents of their corresponding extractable fractions. However, in S-7, caffeic acid appeared relatively higher content in the free fraction of the phenolics. In S-8, there were no bound form caffeic acid or protocatechuic acid detected. Meanwhile, the contents of free form *p*-coumaric were higher than its bound form.

Ferulic acid has been reported as the most abundant bound phenolic compound in sorghum [22] and other cereals [8]. The contents of ferulic acid in bound fractions in the investigated sorghum grains also showed the highest level compared to other phenolic acids, accounted for over 60% of the total identified phenolic acids and ranged from 1.55 ± 0.11 to 85.98 ± 2.72 mg/100 g grain (DW). In the present study, the concentration of ferulic acid in S-7 was higher than the reported levels in sorghum grains, oat, rice, wheat and barley [8,17,23].

Total concentration of caffeic acid varied from 3.49 ± 0.13 to 8.17 ± 0.20 mg/100 g grain (DW), lowest in S-8 and highest in S-1. In S-8, caffeic acid was only detected in free fraction of phenolics. Similar levels were reported before in other varieties of sorghums [15,17,22].

The contents of *p*-coumaric acid and protocatechuic acid ranged from 1.51 ± 0.05 to 8.17 ± 0.28 mg/100 g grain (DW) and 0.57 ± 0.06 to 11.87 ± 0.03 mg/100 g grain (DW) in each total concentration, respectively. Protocatechuic acid was the only benzoic acid derivative in the studied sorghum varieties and S-1 showed the highest concentration in both free and bound fraction of phenolics. These two phenolic acids were not distinct in some sorghum varieties [17,22,24], but they showed abundant concentration in the samples investigated.

Sorghum contained a wide range of flavonoid compounds, and in this study, a total of six flavonoids were quantified, including two 3-deoxyanthocyanidins, two flavones, one dihydroflavonol and one flavanone.

The most common anthocyanins in sorghum are the 3-deoxyanthocyanidins, which include luteolinidin and apigeninidin. These anthocyanins have a small distribution in nature and are special for their lacking of a hydroxyl group at the C-3 position and exist in nature substantially as aglycones [25]. The contents of apigeninidin among all detected sorghum grains were higher than the content of luteolinidin. Brewing sorghum grains and seed-reserved sorghum grains both had high levels of apigeninidin, with the total content ranging from 2.89 ± 0.12 to 4.77 ± 0.29 mg/100g grain (DW). It is reported that sorghums with a black pericarp have the highest levels of 3-deoxyanthocyanidins, which are concentrated in the bran [26]. However, in the investigated black grain sorghum (S-7), the total contents of 3-deoxyanthocyanidins were less than other six red/brown grain sorghums except S-8, which was white grain sorghum. Therefore, the contents of 3-deoxyanthocyanidins in sorghum grains differ from the rule in bran.

Luteolin and apigenin were the two flavones identified in this study. The flavones content in free and bound fractions varied. Only in S-8, free form flavones showed high contents than their bound form. Among all varieties, S-7 had the highest contents of total luteolin with 4.98 ± 0.07 mg/100 g grain (DW) while S-8 had the highest contents of total apigenin with 3.97 ± 0.31 mg/100 g grain (DW). Among four brewing sorghum grains, S-1 showed the relatively high contents of flavones.

Taxifolin was the unique dihydroflavonol detected in the investigated sorghums, which was also the main flavonoid existed in brewing sorghum and seed-reserved sorghum grains. The contents of total taxifolin of above sorghum varieties ranged from 1.37 ± 0.01 to 44.62 ± 2.37 mg/100 g grain (DW), highest in S-1 and lowest in S-4, which accounted for 88% and 26% of the total content of the flavonoids investigated, respectively. And the contents in free fractions were much higher than contents in corresponding bound fractions except S-4. In addition, the content of free forms of taxifolin was extremely high in S-1, which may be a reason causing its specialty from other brewing sorghums.

The flavanone naringenin was analyzed in this study. Naringenin was mainly detected in free forms, the concentration of which ranged from ND to 1.18 mg/100 g grain (DW). There was no free form naringenin detected in S-2, S-3, S-5, S-7 and S-8. And in S-7, no bound form naringenin was detected, either.

### 3.3. Antioxidant Capacities and Their Correlation with Phenolic Compounds

Antioxidants are widely acknowledged for their inhibition or delay to the oxidation of an oxidizable substrate in a chain reaction, which would be very important in the prevention of various physiological and pathological abnormalities, such as inflammation, cardiovascular diseases and ageing [27]. Therefore, antioxidant capacity of sorghum grains with different applications was determined and two methods were used with different principles, including the DPPH assay and the FRAP assay. DPPH is a stable free radical and will accept an electron or a hydrogen atom when free radical scavenger exists, which will result a color change [28]. FRAP assay assesses the antioxidant power by a reduction from ferric to ferrous ion at low pH and causes a colored ferrous-tripyridyltriazine complex to form [29]. All values of free, bound and total phenolic fractions were expressed as milligrams of vitamin C equivalent per gram of grains and were shown in Table 5. The correlations between antioxidant capacities and phenolic compounds were shown in Figure 2. The antioxidant capacity of free fractions among all varieties showed significant differences (*p* < 0.05), ranged from 0.90 ± 0.01 to 18.43 ± 0.88 mg/g VcE (DW) and 2.02 ± 0.05 to 11.66 ± 0.39 mg/g VcE (DW) by the DPPH and FRAP assay, respectively. On the whole, S-1 showed the strongest antioxidant activity, followed by S-3 and weakest in S-8.

The values of in vitro antioxidant properties had strong correlation with the total phenolics of free and bound fractions (*r* > 0.95, *p* < 0.01), which may be caused by the condensed tannins detected in the investigated sorghums (*r* > 0.96, *p* < 0.01) [30]. However, the correlation between TFC and antioxidant capacity was not so strong, which may be caused by the flavonoids in sorghum grains, especially apigenin and naringenin (*r* < 0.5) with one hydroxyl group in the B ring [31,32].

Among all the individual phenolic compounds detected, free fractions of protocatechuic acid and taxifolin had considerably strong correlation with in vitro antioxidant properties, which may explain the highest antioxidant capacity in S-1. It was reported that taxifolin played a special role in maintaining normal functions of circulatory system because of its unique antioxidant activity [33]. And protocatechuic acid was reported to exhibited dose-dependently antioxidant ability which may be closely correlated with various pharmacological effects [34].

## 4. Conclusions

Comparing the differences in phenolic compositions and antioxidant capacities of different varieties of sorghum grains are reported for the first time in the present study. Brewing sorghums grains showed high phenolic, proanthocyanidins and flavonoids contents, as well as in vitro antioxidant, especially S-1. All of the sorghum grains contained high contents of phenolic acids, especially bound form ferulic acid. Most sorghum grains contained various flavonoids, and taxifolin was the dominant one in free fraction extracts. Protocatechuic acid and taxifolin showed considerably strong correlation with in vitro antioxidant properties and exhibited relatively high contents in brewing sorghum grains. These results indicate that brewing sorghum grains can also be utilized as effective materials for functional foods to improve public health. Meanwhile, further work could be proceeded to explore the role of phytochemicals during making distilled liquor and the variation of their antioxidant activities.

## Figures and Tables

**Table 1:** The geographic information of the analyzed sorghum grains.

Environmental Conditions	S-1	S-2	S-3	S-4	S-5~S-8
Locality	Renhuai City	Luzhou City	Jilin City	Suqian City	Jinan City
Latitude	27°69′ N	27°41′~28°20′ N	40°50′~46°19′ N	33°96′ N	36°40′ N
Longitude	106°06′ E	105°34′~106°20′ E	121°38′~131°19′ E	118°30′ E	117°00′ E
Altitude (average)/meter	880	250	196	40	57.8
Climate regime	Subtropical humid monsoon climate	Subtropical monsoon humid climate	Temperate monsoon climate	Warm temperate to subtropical transitional climate	Temperate monsoon climate
Cultivation method	The field planting				

**Table 2:** TPC ^1^ (mg GAE ^2^/100 g grain, DW ^3^) ^4^, TFC ^5^ (mg RE ^6^/100 g grain, DW) and TPAC ^7^ (mg CCE ^8^/100 g grain, DW) of free, bound and total fractions of eight sorghum varieties.

**Varieties**		**S-1**	**S-2**	**S-3**	**S-4**
TPC	Free	1051.84 ± 32.57 ^f^ (84.91%)	473.74 ± 2.56 ^d^ (70.89%)	940.60 ± 33.34 ^e^ (84.42%)	193.83 ± 8.64 ^a^ (59.67%)
	Bound	186.99 ± 7.99 ^de^ (15.09%)	194.55 ± 5.29 ^e^ (29.11%)	173.63 ± 9.10 ^cd^ (15.58%)	131.03 ± 7.50 ^b^ (40.33%)
	Total	1238.83 ± 31.67 ^g^	668.30 ± 7.64 ^e^	1114.23 ± 27.86 ^f^	324.85 ± 13.70 ^b^
TFC	Free	39.80 ± 6.62 ^d^ (65.13%)	12.91 ± 2.61 ^ab^ (63.97%)	18.91 ± 4.50 ^bc^ (72.66%)	15.94 ± 3.96 ^abc^ (46.18%)
	Bound	21.30 ± 1.22 ^g^ (34.87%)	7.27 ± 1.04 ^c^ (36.03%)	7.11 ± 0.60 ^c^ (27.34%)	18.58 ± 0.29 ^f^ (53.82%)
	Total	61.10 ± 5.46 ^d^	20.18 ± 3.65 ^b^	26.03 ± 3.91 ^b^	34.52 ± 3.72 ^c^
TPAC	Free	892.67 ± 31.84 ^d^	266.49 ± 9.88 ^c^	887.17 ± 59.27 ^d^	6.60 ± 1.55 ^a^
**Varieties**		**S-5**	**S-6**	**S-7**	**S-8**
TPC	Free	402.81 ± 35.34 ^c^ (71.99%)	453.92 ± 45.81 ^d^ (70.85%)	251.68 ± 7.01 ^b^ (60.94%)	171.40 ± 4.09 ^a^ (98.07%)
	Bound	156.70 ± 15.54 ^c^ (28.01%)	186.75 ± 10.29 ^de^ (29.15%)	161.29 ± 16.28 ^c^ (39.06%)	3.37 ± 0.57 ^a^ (1.93%)
	Total	559.50 ± 50.39 ^d^	640.67 ± 54.87 ^e^	412.98 ± 9.20 ^c^	174.40 ± 4.09 ^a^
TFC	Free	21.97 ± 3.51 ^c^ (58.00%)	10.08 ± 1.97 ^a^ (51.29%)	18.43 ± 1.87 ^bc^ (87.17%)	11.18 ± 1.65 ^a^ (95.39%)
	Bound	15.91 ± 0.41 ^e^ (42.00%)	9.58 ± 0.97 ^d^ (48.71%)	2.71 ± 0.43 ^b^ (12.83%)	0.54 ± 0.06 ^a^ (4.61%)
	Total	37.88 ± 3.81 ^c^	19.66 ± 2.40 ^b^	21.14 ± 1.80 ^b^	11.72 ± 1.69 ^a^
TPAC	Free	186.24 ± 3.03 ^b^	264.35 ± 15.11 ^c^	17.60 ± 4.05 ^a^	2.47 ± 0.59 ^a^

^1^ TPC means total phenolic contents; ^2^ GAE means gallic acid equivalents; ^3^ DW means dry weight; ^4^ Values are means of triplicates ± standard deviation; ^5^ TFC means total flavonoid contents; ^6^ RE means rutin equivalents; ^7^ TPAC means total proanthocyanidins contents; ^8^ CCE means cyanidin chloride equivalents; ^a–g^ Values with different superscripts in the same row are significantly different (*p* ≤ 0.05).

**Table 3:** The extractable and bound individual phenolic contents (mg/100 g grain, DW ^1^) ^2^ in eight varieties of sorghum grains.

Phenolics	Fraction	S-1	S-2	S-3	S-4	S-5	S-6	S-7	S-8
**Phenolic Acids**									
**Hydrocinnamic Acid**									
Caffeic acid	Free	3.81 ± 0.10 ^f^	2.49 ± 0.13 ^d^	1.14 ± 0.02 ^a^	1.87 ± 0.06 ^c^	1.59 ± 0.24 ^b^	1.61 ± 0.14 ^b^	2.38 ± 0.13 ^d^	3.49 ± 0.13 ^e^
	Bound	4.36 ± 0.12 ^d^	4.40 ± 0.14 ^d^	3.08 ± 0.07 ^c^	4.71 ± 0.04 ^e^	1.92 ± 0.11 ^b^	5.17 ± 0.13 ^f^	2.03 ± 0.12 ^b^	ND ^a3^
	Total	8.17 ± 0.20 ^d^	6.90 ± 0.28 ^c^	4.22 ± 0.09 ^b^	6.58 ± 0.10 ^c^	3.52 ± 0.35 ^a^	6.78 ± 0.28 ^c^	4.41 ± 0.03 ^b^	3.49 ± 0.13 ^a^
*p*-Coumaric acid	Free	0.82 ± 0.08 ^b^	3.08 ± 0.06 ^f^	0.96 ± 0.01 ^c^	1.18 ± 0.06 ^d^	1.37 ± 0.12 ^e^	0.53 ± 0.01 ^a^	0.52 ± 0.01 ^a^	1.05 ± 0.04 ^c^
	Bound	7.35 ± 0.21 ^d^	4.79 ± 0.09 ^b^	5.91 ± 0.62 ^c^	4.57 ± 0.20 ^b^	4.60 ± 0.13 ^b^	7.38 ± 0.74 ^d^	7.17 ± 0.58 ^d^	0.46 ± 0.01 ^a^
	Total	8.17 ± 0.28 ^d^	7.87 ± 0.04 ^d^	6.87 ± 0.61 ^c^	5.75 ± 0.17 ^b^	5.97 ± 0.24 ^b^	7.91 ± 0.75 ^d^	7.69 ± 0.59 ^d^	1.51 ± 0.05 ^a^
Ferulic acid	Free	2.68 ± 0.06 ^e^	1.45 ± 0.08 ^d^	1.02 ± 0.05 ^c^	0.78 ± 0.01 ^b^	0.96 ± 0.07 ^c^	0.67 ± 0.01 ^a^	0.86 ± 0.02 ^b^	0.85 ± 0.02 ^b^
	Bound	39.66 ± 1.26 ^c^	42.72 ± 0.35 ^d^	38.67 ± 0.59 ^c^	56.84 ± 1.06 ^e^	33.25 ± 1.09 ^b^	33.06 ± 1.01 ^b^	85.98 ± 2.72 ^f^	1.55 ± 0.11 ^a^
	Total	42.34 ± 1.19 ^d^	44.18 ± 0.28 ^d^	39.69 ± 0.64 ^c^	57.62 ± 1.05 ^e^	34.21 ± 1.02 ^b^	33.73 ± 1.00 ^b^	86.84 ± 2.74 ^f^	2.40 ± 0.10 ^a^
**Hydrobenzoic acids**									
Protocatechuic acid	Free	5.31 ± 0.16 ^e^	2.20 ± 0.35 ^c^	4.01 ± 0.12 ^d^	0.58 ± 0.02 ^a^	1.66 ± 0.04 ^b^	1.52 ± 0.04 ^b^	0.56 ± 0.04 ^a^	0.57 ± 0.06 ^a^
	Bound	6.56 ± 0.15 ^g^	5.99 ± 0.31 ^f^	5.36 ± 0.39 ^e^	1.10 ± 0.02 ^b^	2.91 ± 0.27 ^c^	4.92 ± 0.30 ^d^	1.27 ± 0.06 ^b^	ND ^a^
	Total	11.87 ± 0.03 ^g^	8.19 ± 0.05 ^e^	9.36 ± 0.37 ^f^	1.69 ± 0.03 ^b^	4.57 ± 0.30 ^c^	6.44 ± 0.34 ^d^	1.84 ± 0.10 ^b^	0.57 ± 0.06 ^a^
**Flavonoids**									
**3-Deoxyanthocyanidin**									
Luteolinidin	Free	0.67 ± 0.02 ^d^	0.37 ± 0.00 ^b^	0.43 ± 0.01 ^c^	1.27 ± 0.01 ^e^	1.67 ± 0.04 ^f^	ND ^a^	ND ^a^	ND ^a^
	Bound	0.84 ± 0.02 ^e^	0.62 ± 0.02 ^c^	ND ^a^	ND ^a^	0.80 ± 0.01 ^d^	0.50 ± 0.02 ^b^	ND ^a^	ND ^a^
	Total	1.51 ± 0.00 ^f^	0.99 ± 0.02 ^d^	0.43 ± 0.01 ^b^	1.27 ± 0.01 ^e^	2.47 ± 0.05 ^g^	0.50 ± 0.02 ^c^	ND ^a^	ND ^a^
Apigeninidin	Free	3.06 ± 0.29 ^f^	1.55 ± 0.04 ^d^	1.16 ± 0.01 ^c^	1.53 ± 0.01 ^d^	2.13 ± 0.17 ^e^	0.86 ± 0.02 ^b^	0.71 ± 0.03 ^b^	ND ^a^
	Bound	1.70 ± 0.02 ^c d^	1.60 ± 0.03 ^c^	2.10 ± 0.22 ^e^	2.55 ± 0.27 ^f^	1.29 ± 0.03 ^b^	2.03 ± 0.14 ^e^	1.91 ± 0.12 ^d e^	0.70 ± 0.02 ^a^
	Total	4.77 ± 0.29 ^f^	3.16 ± 0.02 ^c d^	3.26 ± 0.22 ^d^	4.07 ± 0.28 ^e^	3.43 ± 0.16 ^d^	2.89 ± 0.12 ^b c^	2.62 ± 0.14 ^b^	0.70 ± 0.02 ^a^
**Flavones**									
Luteolin	Free	1.95 ± 0.12 ^d^	1.02 ± 0.01 ^b^	0.95 ± 0.01 ^b^	0.68 ± 0.03 ^a^	1.11 ± 0.02 ^b c^	0.70 ± 0.02 ^a^	3.60 ± 0.24 ^e^	1.21 ± 0.09 ^c^
	Bound	0.92 ± 0.02 ^d^	0.64 ± 0.00 ^b^	0.78 ± 0.02 ^c^	0.94 ± 0.02 ^d^	0.79 ± 0.02 ^c^	0.90 ± 0.02 ^d^	1.38 ± 0.17 ^e^	ND ^a^
	Total	2.87 ± 0.10 ^e^	1.66 ± 0.02 ^b c^	1.73 ± 0.01 ^c^	1.63 ± 0.02 ^b^	1.91 ± 0.00 ^d^	1.61 ± 0.01 ^b^	4.98 ± 0.07 ^f^	1.21 ± 0.09 ^a^
Apigenin	Free	1.21 ± 0.06 ^b^	0.30 ± 0.01 ^a^	1.20 ± 0.03 ^b^	0.47 ± 0.02 ^a^	0.45 ± 0.03 ^a^	0.40 ± 0.01 ^a^	0.30 ± 0.01 ^a^	3.62 ± 0.32 ^c^
	Bound	0.66 ± 0.01 ^e^	0.37 ± 0.02 ^b^	0.50 ± 0.01 ^c^	0.52 ± 0.01 ^c^	0.55 ± 0.01 ^d^	0.32 ± 0.01 ^a^	0.64 ± 0.02 ^e^	0.34 ± 0.01 ^a^
	Total	1.87 ± 0.06 ^c^	0.67 ± 0.03 ^a^	1.70 ± 0.02 ^c^	0.98 ± 0.01 ^b^	0.99 ± 0.04 ^b^	0.72 ± 0.02 ^a^	0.94 ± 0.00 ^b^	3.97 ± 0.31 ^d^
**Dihydroflavonol**									
Taxifolin	Free	42.98 ± 2.33 ^d^	9.33 ± 0.16 ^c^	5.77 ± 0.12 ^b^	0.60 ± 0.01 ^a^	6.05 ± 0.42 ^b^	9.22 ± 0.87 ^c^	ND ^a^	0.53 ± 0.01 ^a^
	Bound	1.65 ± 0.04 ^e^	1.04 ± 0.01 ^c^	1.07 ± 0.01 ^c^	0.77 ± 0.02 ^b^	1.75 ± 0.17 ^e^	1.32 ± 0.15 ^d^	ND ^a^	ND ^a^
	Total	44.62 ± 2.37 ^d^	10.36 ± 0.15 ^c^	6.83 ± 0.11 ^b^	1.37 ± 0.01 ^a^	7.80 ± 0.58 ^b^	10.53 ± 0.73 ^c^	ND ^a^	0.53 ± 0.01 ^a^
**Flavanone**									
Naringenin	Free	0.72 ± 0.02 ^c^	1.18 ± 0.12 ^f^	0.47 ± 0.01 ^b^	0.86 ± 0.01 ^d^	0.50 ± 0.04 ^b^	0.44 ± 0.02 ^b^	ND ^a^	0.98 ± 0.03 ^e^
	Bound	0.66 ± 0.02 ^d^	ND ^a^	ND ^a^	0.33 ± 0.00 ^b^	ND ^a^	0.51 ± 0.02 ^c^	ND ^a^	ND ^a^
	Total	1.38 ± 0.04 ^e^	1.18 ± 0.12 ^d^	0.47 ± 0.01 ^b^	1.19 ± 0.01 ^d^	0.50 ± 0.04 ^b^	0.95 ± 0.04 ^c^	ND ^a^	0.98 ± 0.03 ^c^

^1^ DW means dry weight. ^2^ Values are means of triplicates ± standard deviation. ^a–g^ Values with different superscripts in the same row are significantly different (*p* ≤ 0.05). ^3^ ND, data not detected.

**Table 4:** Identification of phenolic compounds in sorghum grains ^1^ by RP-UHPLC-QTOF/MS ^2^.

Peak No.	Rt^2^ (min)	[M − H]^−^	[MS/MS]	Tentative Identification	Formula
1	11.82	153	65, 83, 123, 151	Protocatechuic acid	C_7_H_6_O_4_
2	15.79	179	89, 105, 133, 149, 177	Caffeic acid	C_9_H_8_O_4_
3	16.74	270	133, 182, 99, 225, 270	Luteolinidin	C_15_H_11_O_5_^+^
4	19.29	254	118, 181, 211, 254	Apigeninidin	C_15_H_11_O_4_^+^
5	21.20	163	104, 115, 133	*p*-coumaric acid	C_9_H_8_O_3_
6	22.16	193	137, 193	Ferulic acid	C_10_H_10_O_4_
7	24.41	303	125, 150, 175, 199, 217, 241, 285	Taxifolin	C_15_H_12_O_7_
8	33.85	285	133, 285	Luteolin	C_15_H_10_O_6_
9	39.37	269	177, 269	Apigenin	C_15_H_10_O_5_
10	39.94	271	119, 151, 177	Naringenin	C_15_H_12_O_5_

^1^ The phenolic extract of S-1 was used to identify the individual compounds. ^2^ The RP-UPLC-QTOF/MS was detected under 288 nm.

**Table 5:** Antioxidant capacity (mg VcE ^1^/g grain, DW ^2^) ^3^ of free and bound phenolic extract measured by 2,2-diphenyl-1-picrylhydrazyl radicals (DPPH) and ferric reducing ability of plasma (FRAP) assays.

**Antioxidant Capacity**	**Fraction**	**S-1**	**S-2**	**S-3**	**S-4**
DPPH	Free	18.43 ± 0.88 ^f^ (96.76%)	4.62 ± 0.10 ^d^ (87.11%)	14.41 ± 0.94 ^e^ (96.39%)	1.20 ± 0.12 ^ab^ (74.46%)
	Bound	0.62 ± 0.02 ^e^ (3.24%)	0.68 ± 0.02 ^f^ (12.89%)	0.54 ± 0.02 ^c^ (3.61%)	0.41 ± 0.03 ^b^ (25.54%)
	Total	19.05 ± 0.86 ^f^	5.31 ± 0.09 ^d^	14.94 ± 0.92 ^e^	1.61 ± 0.14 ^ab^
FRAP	Free	11.66 ± 0.39 ^e^ (85.83%)	3.99 ± 0.09 ^c^ (65.14%)	8.87 ± 0.81 ^d^ (81.41%)	2.22 ± 0.11 ^ab^ (59.97%)
	Bound	1.92 ± 0.12 ^d^ (14.17%)	2.14 ± 0.04 ^e^ (34.86%)	2.03 ± 0.05 ^d^ (18.59%)	1.48 ± 0.03 ^b^ (40.03%)
	Total	13.59 ± 0.47 ^f^	6.13 ± 0.13 ^d^	10.90 ± 0.78 ^e^	3.71 ± 0.13 ^b^
**Antioxidant Capacity**	**Fraction**	**S-5**	**S-6**	**S-7**	**S-8**
DPPH	Free	2.95 ± 0.02 ^c^ (83.26%)	4.32 ± 0.14 ^d^ (88.49%)	1.73 ± 0.06 ^b^ (73.73%)	0.90 ± 0.01 ^a^ (97.93%)
	Bound	0.59 ± 0.02 ^de^ (16.74%)	0.56 ± 0.03 ^cd^ (11.51%)	0.62 ± 0.05 ^e^ (26.27%)	0.02 ± 0.01 ^a^ (2.07%)
	Total	3.55 ± 0.03 ^c^	4.89 ± 0.16 ^d^	2.35 ± 0.10 ^b^	0.92 ± 0.03 ^a^
FRAP	Free	3.85 ± 0.02 ^c^ (68.66%)	3.77 ± 0.04 ^c^ (63.84%)	2.64 ± 0.05 ^b^ (60.64%)	2.02 ± 0.05 ^a^ (82.85%)
	Bound	1.76 ± 0.03 ^c^ (31.34%)	2.14 ± 0.10 ^e^ (36.16%)	1.71 ± 0.03 ^c^ (39.36%)	0.42 ± 0.01 ^a^ (17.25%)
	Total	5.61 ± 0.05 ^d^	5.91 ± 0.14 ^d^	4.35 ± 0.06 ^c^	2.44 ± 0.05 ^a^

^1^ VcE means ascorbic acid equivalents. ^2^ DW means dry weight. ^3^ Values are means of triplicates ± standard deviation. ^a–f^ Values with different superscripts in the same fraction of the same assay are significantly different (*p* ≤ 0.05).

## Supplementary Materials

The following are available online. Figures S1–S10: MS/MS spectra of individual phenolic compounds in negative electrospray ionization.

## Abbreviations

The following abbreviations are used in this manuscript:

TPC	Total phenolic contents
TFC	Total flavonoid contents
TPAC	Total proanthocyanidin contents
GAE	Gallic acid equivalents
RE	Rutin equivalents
CCE	Cyanidin chloride equivalents
DW	Dry weight
ND	Data not detected
DPPH	Diphenylpicrylhydrazyl
FRAP	Ferric reducing ability of plasma
