# Supplementary material for: Phenolic Compositions and Antioxidant Activities Differ Significantly among Sorghum Grains with Different Applications
Source: Molecules. 2018 May 17;23(5):1203. doi: 10.3390/molecules23051203 (PMC6100422; doi:10.3390/molecules23051203)
Supplement: Supplementary file 1 [file molecules-23-01203-s001.pdf]

Supplementary Materials

Phenolic compositions and antioxidant activities differ significantly among sorghum grains with different applications

Shuyu Shen [1], Rui Huang [1], Charlie Li [2], WenYan Wu [1], Honglin Chen [1], John Shi [3,*], Shiguo Chen [1,*] and Xingqian Ye [1,*]

1. Zhejiang University, College of Biosystem Engineering and Food Science, Zhejiang Key Laboratory for Agro-Food Processing, Fuli Institute of Food Science, Hangzhou 310058, China;
2. University of California-Davis, Department of Environmental Toxicology, Davis, CA 94564, USA;
3. Guelph Research and Development Center, Agriculture and Agri-Food Canada, Guelph, Ontario, Canada;

* Correspondence: johnshi2006@yahoo.ca; chenshiguo210@163.com, Tel.: 86-571-88982151.; psu@zju.edu.cn, Tel.: 86-571-88982155

Figure S1. MS/MS spectra of Protocatechuic acid in negative electrospray ionization

Figure S2. MS/MS spectra of Caffeic acid in negative electrospray ionization

Figure S3. MS/MS spectra of Luteolinidin in negative electrospray ionization

Figure S4. MS/MS spectra of Apigeninidin in negative electrospray ionization

Figure S5. MS/MS spectra of *p*-coumaric acid in negative electrospray ionization

Figure S6. MS/MS spectra of Ferulic acid in negative electrospray ionization

Figure S7. MS/MS spectra of Taxifolin in negative electrospray ionization

Figure S8. MS/MS spectra of Luteolin in negative electrospray ionization

Figure S9. MS/MS spectra of Apigenin in negative electrospray ionization

Figure S10. MS/MS spectra of Narigenin in negative electrospray ionization

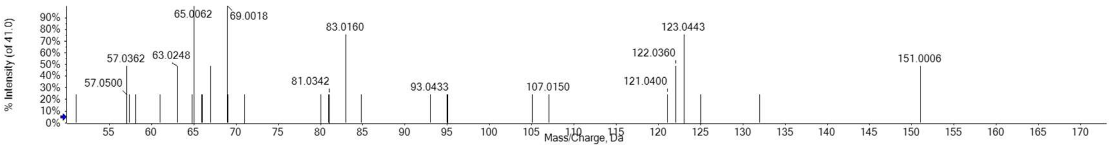

Figure S1. MS/MS spectra of Protocatechuic acid in negative electrospray ionization

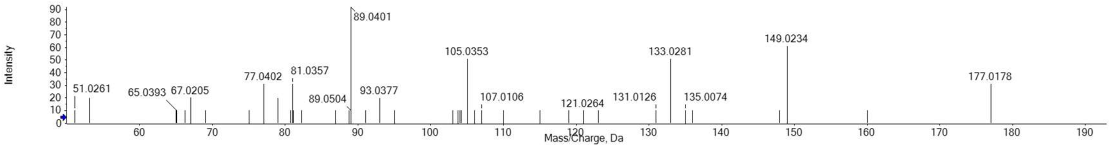

Figure S2. MS/MS spectra of Caffeic acid in negative electrospray ionization

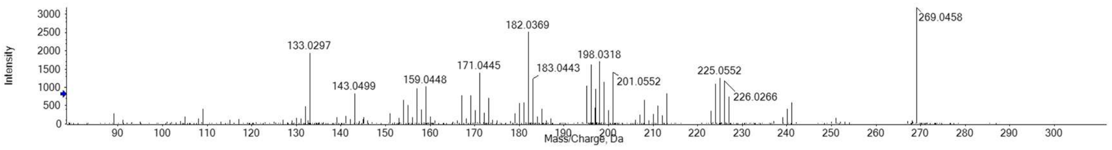

Figure S3. MS/MS spectra of Luteolinidin in negative electrospray ionization

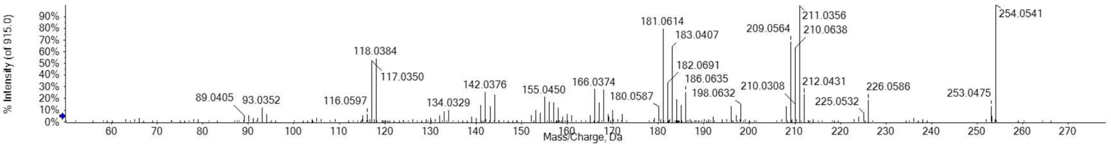

Figure S4. MS/MS spectra of Apigeninidin in negative electrospray ionization

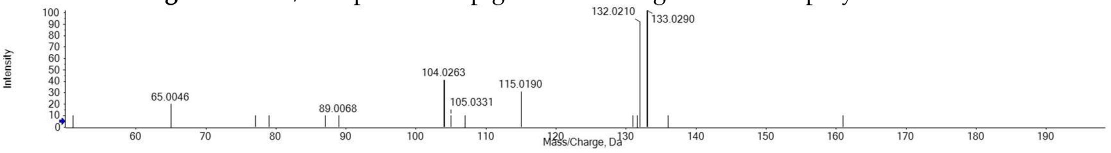

Figure S5. MS/MS spectra of *p*-coumaric acid in negative electrospray ionization

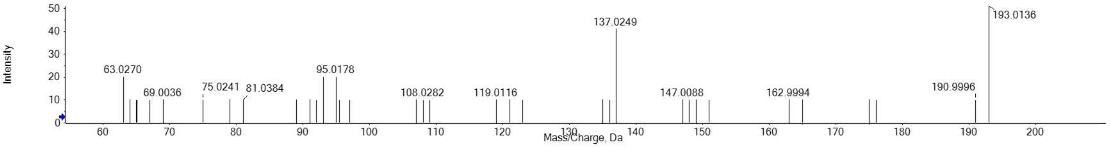

Figure S6. MS/MS spectra of Ferulic acid in negative electrospray ionization

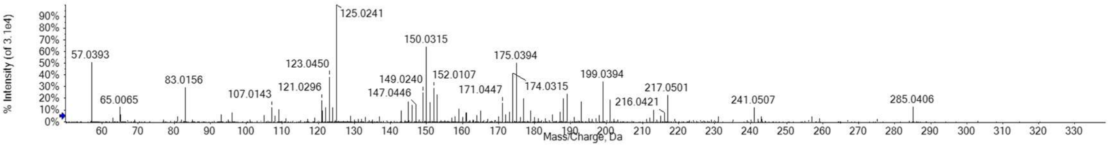

Figure S7. MS/MS spectra of Taxifolin in negative electrospray ionization

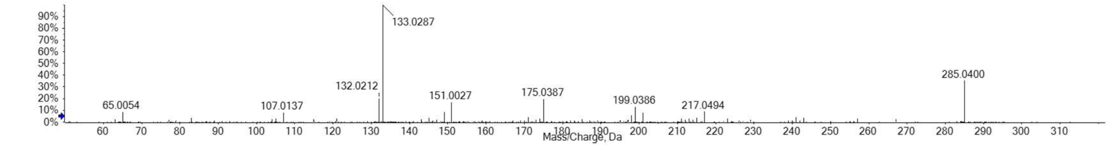

Figure S8. MS/MS spectra of Luteolin in negative electrospray ionization

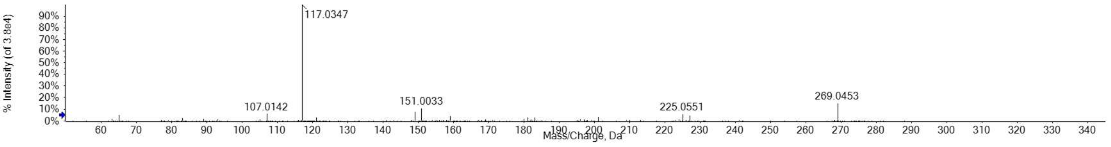

Figure S9. MS/MS spectra of Apigenin in negative electrospray ionization

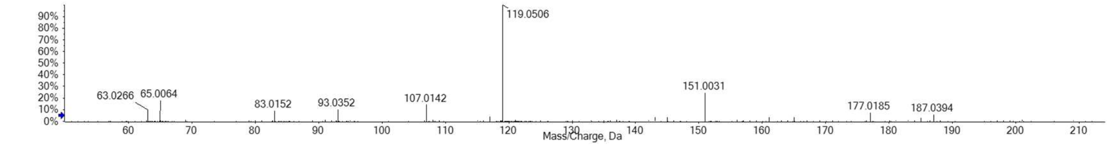

Figure S10. MS/MS spectra of Narigenin in negative electrospray ionization
